# Primary extranodal marginal zone lymphoma of mucosa-associated lymphoid tissue with multiple pure ground-glass opacities: a case report

**DOI:** 10.1186/s13019-017-0565-9

**Published:** 2017-01-25

**Authors:** Xuebing Ding, Takashi Makino, Satoshi Koezuka, Takashi Azumi, Hajime Otsuka, Yoshinobu Hata, Yuichi Shinya, Naobumi Tochigi, Kazutoshi Shibuya, Akira Iyoda

**Affiliations:** 1 Division of Chest Surgery, Toho University School of Medicine, Tokyo, Japan; 2 Department of Cardiothoracic Surgery, Affiliated Suzhou Hospital of Nanjing Medical University, Suzhou, China; 3 Shinya Clinic, Kanagawa, Japan; 4 Department of Surgical Pathology, Toho University School of Medicine, Tokyo, Japan

**Keywords:** Extranodal marginal zone lymphoma, Mucosa-associated lymphoid tissue, Multiple, Ground-glass opacity

## Abstract

**Background:**

Primary pulmonary mucosa-associated lymphoid tissue (MALT) lymphoma is a low-grade B cell lymphoma that is a type of non-Hodgkin lymphoma and a type of primary pulmonary malignant lymphoma. MALT lymphomas affecting the lung show various findings on chest computed tomography, which range from typical nodules or areas of consolidation to findings that are extremely rare in pulmonary MALT lymphomas, such as pure ground-glass opacities throughout the lung.

**Case presentation:**

A 35-year-old woman was found to have a few shadows with ground glass opacities on chest computed tomography (CT) in 2012. A shadow in right S10 that was initially very small increased in size over time, and was 14 × 8 mm in 2015. Other shadows also appeared. Because lung adenocarcinoma was suspected, the patient underwent video-assisted thoracoscopic surgery with a right wedge resection of the lower lobe that included the largest nodule in S10 and other nodules. Histopathological examination of the right S10 and other lesions revealed small- or medium-sized lymphocyte-like cells that were located in the alveolar interseptal spaces. The alveolar walls remained intact. Immunohistochemical staining showed that tumor cells were positive for CD20, CD79a, and BCL2 expression. The lesions were diagnosed as extranodal marginal zone B-cell lymphoma of MALT.

**Conclusions:**

We think that the ground glass opacities on CT were accounted for by MALT lesions that contained intact alveolar air spaces. The patient has remained well during 12 months of follow up after surgery. Although she did not receive chemotherapy because the MALT lymphoma lesions have been stable without progression, the patient is kept under close observation because of potential progression of the disease.

## Background

Pulmonary extranodal marginal zone lymphoma of mucosa-associated lymphoid tissue (MALT) is a type of primary pulmonary malignant lymphoma with monoclonal B cells, and infiltrates the bronchiolar mucosal epithelium, forming lymphoepithelial lesions [1]. Patients with MALT lymphoma may have nonspecific symptoms and can also be asymptomatic [2]. Chest computed tomography (CT) findings of MALT lymphomas occurring in the lung range from typical nodules or areas of consolidation to findings that are extremely rare, such as pure ground-glass opacities (GGOs) [2]. Here, we report a patient with growth and many GGOs.

## Case presentation

**Fig. 1:**
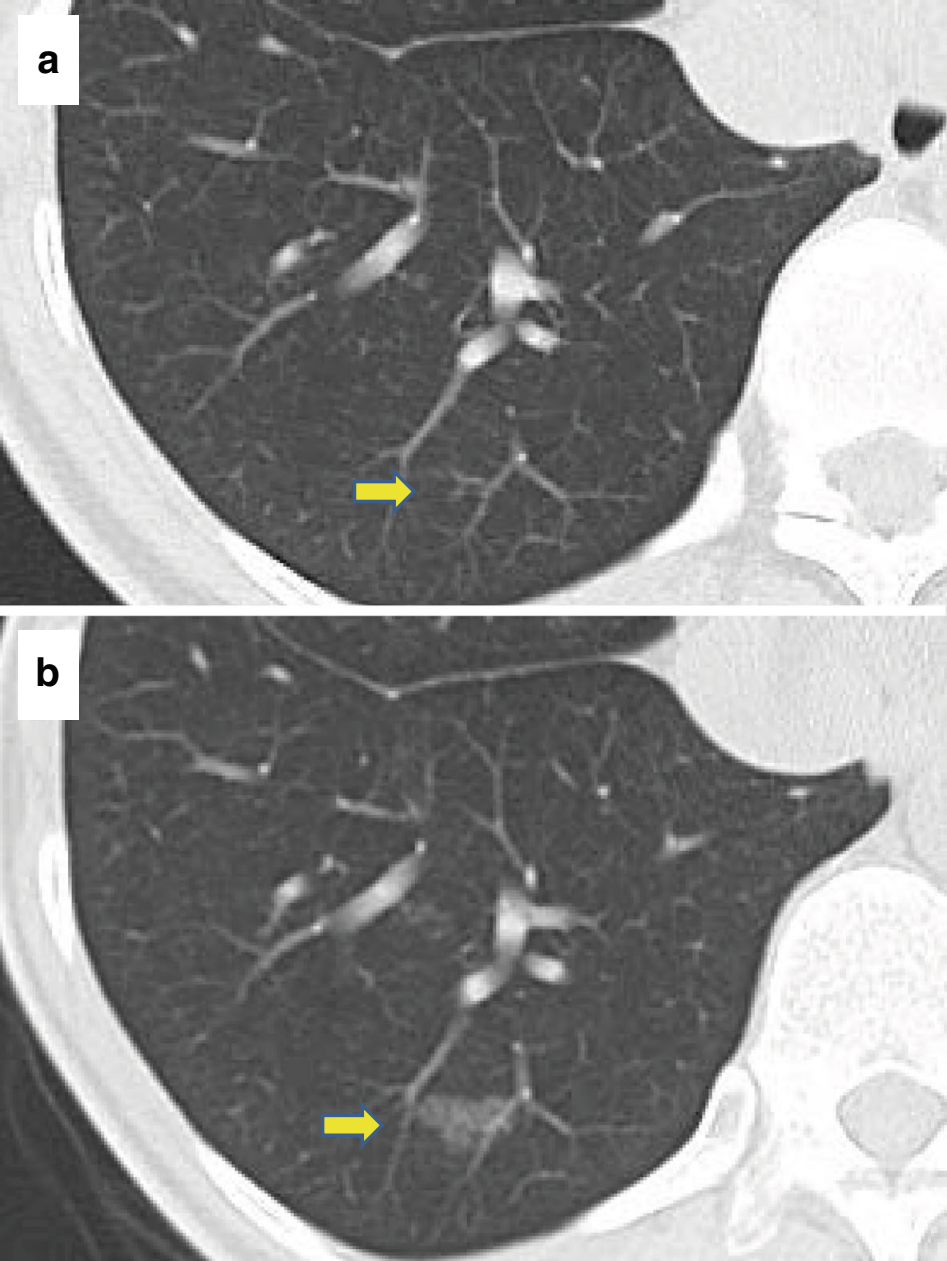
Chest computed tomography in 2012 revealed a very small shadow with ground glass opacity (*arrow*) that increased in size over time. **a** in 2012 (**b**) in 2015

**Fig. 2:**
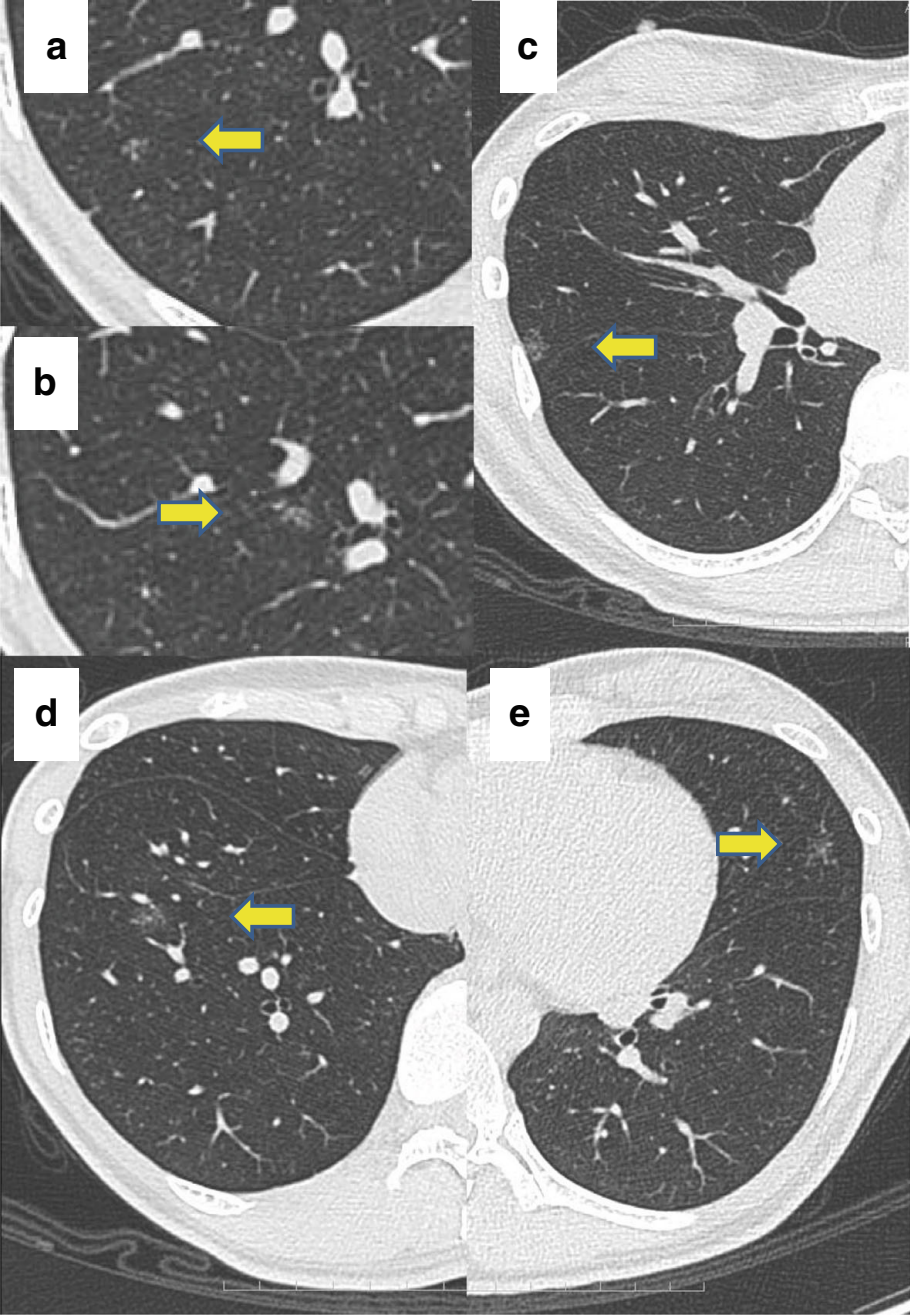
Chest computed tomography in 2015 also revealed multiple shadows with ground glass opacities **a**, **b** in right lower lobe, **c** in right middle lobe, **d** in segment 8 of right lower lobe, and **e** in left upper lobe

A 35-year-old woman was found to have a few shadows with GGOs on chest CT in 2012. She was monitored and a shadow in right S10 was found to increase in size, which suggested lung adenocarcinoma. The patient was referred to our hospital for further evaluation. The patient had a history of bronchial asthma. The shadow in right S10 had increased to 14 × 8 mm by 2015 (Fig. 1). Other shadows also appeared (Fig. 2). After preoperative CT-guided marking, the patient underwent video-assisted thoracoscopic surgery with a right wedge resection of the lower lobe that included the largest nodule in S10 and other nodules. Her postoperative course was uneventful.

**Fig. 3:**
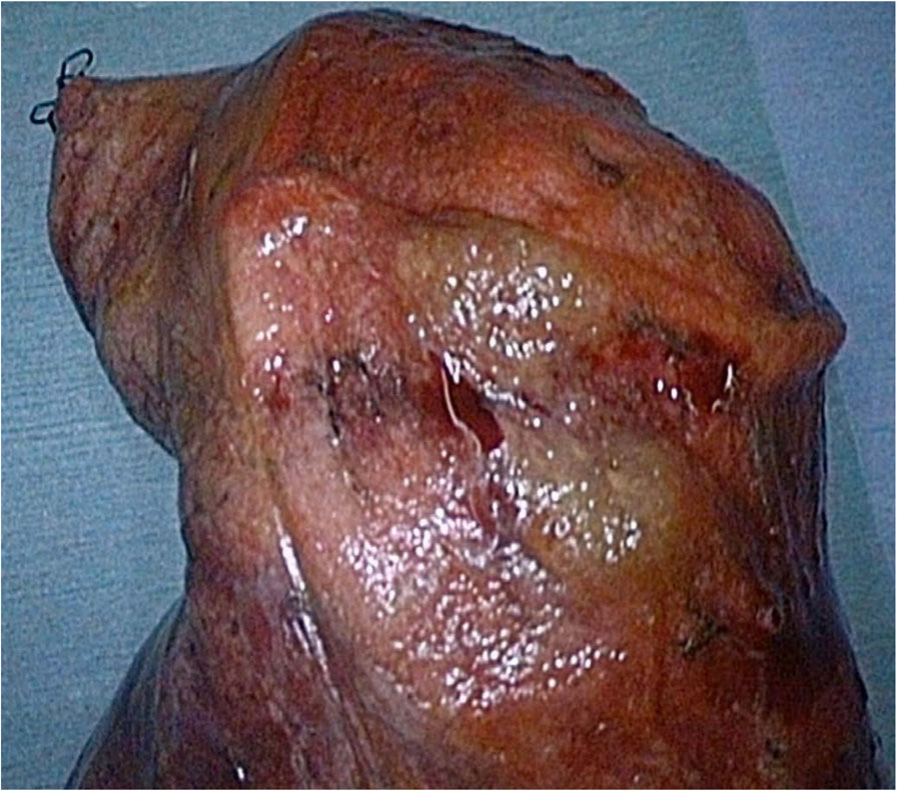
The surgical specimen revealed a yellowish tumor

**Fig. 4:**
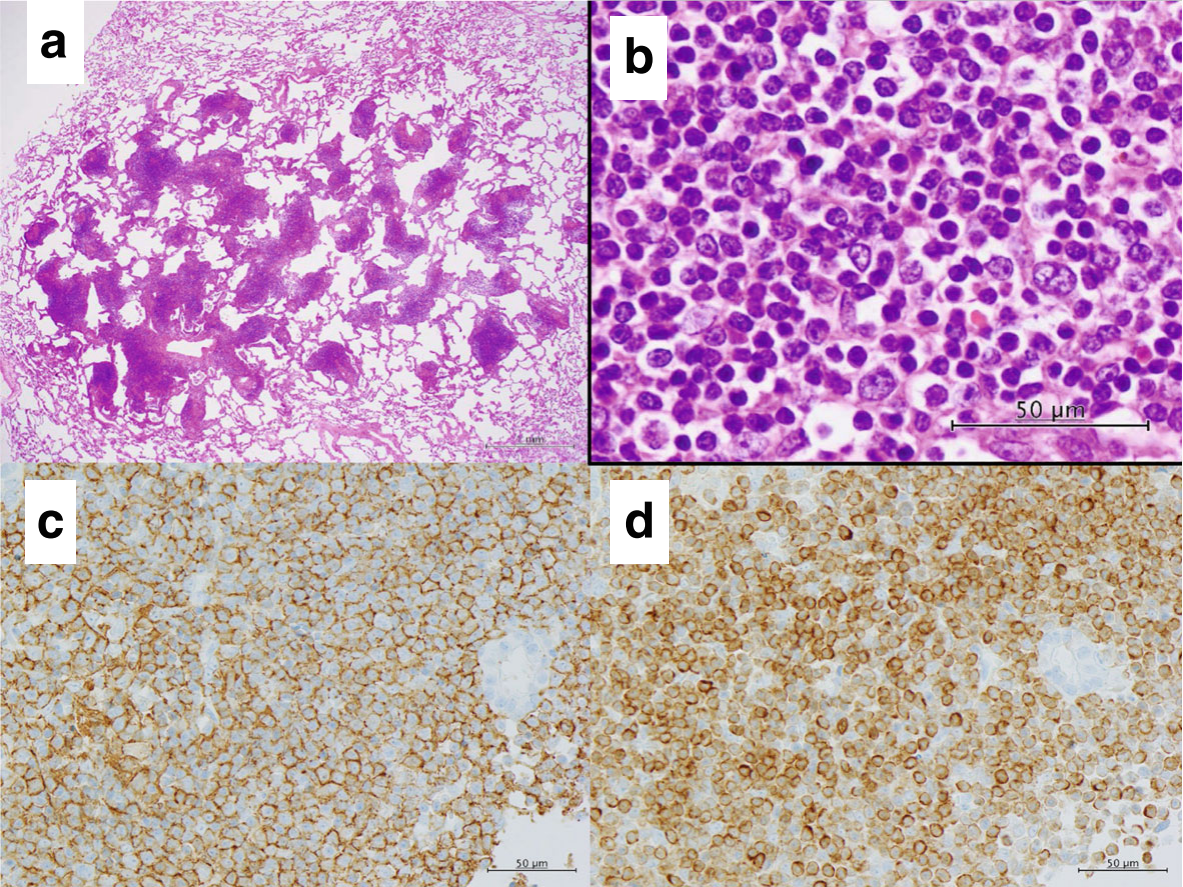
**a** Pathological findings revealed tumor cells that were located in alveolar interseptal spaces. The alveolar walls remained intact. **b** Tumor cells showed slight atypia with hyperchromatic nuclei. Lymphoepithelial lesions were observed in the specimen. **c** Tumor cells were positive for CD20 expression. **d** Tumor cells were positive for BCL2 expression

Grossly, the surgical specimen contained pale, somewhat yellow lesions (Fig. 3). Histopathological examination of the right S10 lesion and other nodules revealed small- or medium-sized lymphocyte-like cells that were located in the alveolar interseptal spaces. The alveolar walls remained intact (Fig. 4a, b). Immunohistochemical staining showed that tumor cells were positive for CD20 (1:400 dilution; DAKO, Carpinteria, CA, USA, Fig. 4c), CD79a (1:200 dilution; DAKO, Carpinteria, CA, USA), and BCL2 (1:50 dilution; DAKO, Carpinteria, CA, USA, Fig. 4d) expression and negative for CD10 (1:50 dilution; Novocastra, Newcastle upon Tyne, UK), cyclin D1 (1:75 dilution; DAKO, Carpinteria, CA, USA) and CD30 (1:40 dilution; DAKO, Carpinteria, CA, USA). The lesions were diagnosed as extranodal marginal zone B-cell lymphoma of MALT. The patient has remained well during 12 months of follow up after surgery. Although she did not receive chemotherapy because the MALT lymphoma lesions have been stable without progression, the patient is kept under close observation because of potential progression of the disease.

## Discussion

Extranodal lymphomas, frequently found in the gastrointestinal tract, occur in less than 5% of patients with Hodgkin lymphoma and up to 33% with non-Hodgkin lymphoma [3].

Primary pulmonary lymphoma is defined as lymphoma with involvement of the lung, lobar, or primary bronchus, with or without mediastinal involvement, and no evidence of extrathoracic lymphoma at the time of diagnosis or for 3 months thereafter [4]. It is an extremely rare neoplasm, accounting for 0.5% of all primary pulmonary malignancies [5], 3–4% of extranodal non-Hodgkin lymphoma, and less than 1% of non-Hodgkin’s lymphoma [6].

In chest CT, the usual finding of primary pulmonary MALT lymphoma is bilateral and multiple, nearly all lesions contain clear areas corresponding to an intact bronchial lumen with sometimes distended bronchi, and less than 10% of patients have bilateral diffuse reticulonodular opacities, atelectasis or pleural effusion [7]. In our patient, the CT lesions appeared as pure GGOs. With the increasing use of low-dose screening CT, increasing numbers of small lung cancers with focal GGOs are being detected [8]. GGO means an area of a homogeneous hazy increase in density that does not obscure the bronchovascular structure in the lung field on thin-section CT [9]. GGOs have been reported in 0.2–0.5% of screened populations [9]. Localized GGOs that persist for months have been reported to correspond to precancerous lesions such as atypical adenomatous hyperplasia and early-stage adenocarcinomas such as bronchioloalveolar carcinoma [9]. Identification and management of persistent pure GGOs that appear on CT are difficult. In our patient, after monitoring her by repeated CT imaging, we interpreted her findings as suspicious for lung adenocarcinoma. However, pathological findings in our case revealed pulmonary extranodal marginal zone B-cell lymphoma of MALT.

Pulmonary MALT lymphoma manifesting as multiple pure GGOs is extremely rare. In our case, we think that GGOs on CT were accounted for by MALT lesions that contained intact alveolar air spaces.

Pulmonary MALT lymphoma usually has an indolent course, remaining localized in the lung for long periods before dissemination [10]. Ahmed et al. studied 22 cases of bronchial-associated lymphoid tissue lymphoma, and they concluded that bronchial-associated lymphoid tissue lymphoma responds well to local or systemic therapy and has a relatively good prognosis [10]. Troch et al. performed follow up without treatment for patients with pulmonary MALT lymphoma. They concluded that it was a very indolent disease, with the potential for spontaneous regression, and that asymptomatic patients with MALT lymphoma might not require immediate treatment [11]. Because our patient had multiple pulmonary lesions, complete excision was difficult; we performed limited thoracoscopic surgical resection for diagnosis only. Instead of surgical excision or chemotherapy for our patient, we are monitoring her other bilateral, slow-growing GGOs. She has remained well during 12 months of follow up after surgery, and all the lesions are stable without progression.

## Conclusions

We reported a case of pulmonary extranodal marginal zone B-cell lymphoma of MALT, with CT features of multiple pure GGOs, which is extremely rare. We think that the ground glass opacities on CT were accounted for by MALT lesions that contained intact alveolar air spaces. The patient has remained well during 12 months of follow up after surgery. Although she did not receive chemotherapy because the MALT lymphoma lesions have been stable without progression, the patient is kept under close observation because of potential progression of the disease.

## Abbreviations

CT: Computed tomography
GGO: Ground-glass opacity
MALT: Mucosa-associated lymphoid tissue
